# Reflections on IDEAL: What we have learnt from a unique calf cohort study

**DOI:** 10.1016/j.prevetmed.2020.105062

**Published:** 2020-08

**Authors:** R. Callaby, A. Jennings, S.T. Mwangi, M. Mbole-Kariuki, I. Van Wyk, H. Kiara, J.A.W. Coetzer, M.E.J. Woolhouse, O. Hanotte, P.G. Toye, B.M. deC. Bronsvoort

**Affiliations:** a Centre for Tropical Livestock Genetics and Health (CTLGH), Easter Bush Campus, EH25 9RG, UK; b The Roslin Institute and Royal (Dick) School of Veterinary Studies, University of Edinburgh, Edinburgh, UK; c Paul G. Allen School for Global Animal Health, Washington State University, USA; d University of Nairobi, Nairobi, Kenya; e African Union Interafrican Bureau for Animal Resources (AU-IBAR), Nairobi, Kenya; f Hans Hoheisen Wildlife Research Station, University of Pretoria, South Africa; g International Livestock Research Institute (ILRI), Nairobi, Kenya; h Department of Veterinary Tropical Diseases, Faculty of Veterinary Science, University of Pretoria, South Africa; i Usher Institute, Deanery of Molecular, Genetic and Population Health Sciences, University of Edinburgh, Edinburgh, UK; j Centre for Tropical Livestock Genetics and Health (CTLGH), ILRI Ethiopia, P.O. Box 5689, Addis Ababa, Ethiopia; k Cells, Organisms and Molecular Genetics, School of Life Sciences, University of Nottingham, Nottingham, UK; l Centre for Tropical Livestock Genetics and Health (CTLGH), ILRI Kenya, P.O. Box 30709, Nairobi, 00100, Kenya

**Keywords:** Cattle, Infectious disease, Kenya, Longitudinal study, Cohort, Epidemiology

## Abstract

The year 2020 marks a decade since the final visit was made in the ‘Infectious Diseases of East African Livestock’ (IDEAL) project. However, data generation from samples obtained during this ambitious longitudinal study still continues. As the project launches its extensive open-access database and biobank to the scientific community, we reflect on the challenges overcome, the knowledge gained, and the advantages of such a project. We discuss the legacy of the IDEAL project and how it continues to generate evidence since being adopted by the Centre for Tropical Livestock Genetics and Health (CTLGH). We also examine the impact of the IDEAL project, from the authors perspective, for each of the stakeholders (the animal, the farmer, the consumer, the policy maker, the funding body, and the researcher and their institution) involved in the project and provide recommendations for future researchers who are interested in running longitudinal field studies.

## 1 Introduction

Constraints on livestock production in Africa are varied and include nutrition, access to markets, natural catastrophes, and importantly infectious diseases (Perry and Grace, 2009). Studies of infectious diseases of livestock in sub-Saharan Africa have most usually focused on single specific infections such as trypanosomiasis or theileriosis / East Coast fever (ECF) which are known to cause serious constraints to farming systems in this region. However, livestock in extensive systems are routinely and simultaneously exposed to a wide variety of pathogens whose impacts on animal health are unlikely to be independent of one another (Petney and Andrews, 1998; Cleaveland et al., 2001). Evidence suggest that pathogens interact to influence the outcome of the infection for the host, or even the epidemiology of infecting pathogens within the host population. This may be by influencing the latency period, or altering the severity of disease and host fitness (Cox, 2001; Lello et al., 2004; Pedersen and Fenton, 2007). These interactions occur through mechanisms such as cross-immunity, immunosuppression, and competition (Lello et al., 2004; Pedersen and Fenton, 2007). For example, in buffalo infection with gastrointestinal worms has been observed to increase bovine tuberculosis infection severity (Ezenwa et al., 2019). It is therefore necessary to study the entire burden of infectious diseases rather than single diseases on their own in order to accurately understand their full impact. The Infectious Diseases of East Africa Livestock (IDEAL) project was conceived to specifically address this problem (Bronsvoort et al., 2013).

The IDEAL project aimed to use a longitudinal cohort study of cattle to address the underlying lack of baseline epidemiological data about infectious disease in Western Kenya and to investigate the concept of a ‘good calf’ by focusing on what combination of a calf’s life history (infectious disease exposure, genetic traits, mothering, husbandry practice, and environmental factors) result in a healthy productive calf, or conversely, a poorly grown or dead calf. In other words, why do some infected individuals suffer very few adverse clinical or productivity impacts, where other infected animals in the same environment are severely affected?

It is now a decade since the final visit was made to the IDEAL project calves and, as samples and data from the IDEAL project are adopted by the Centre for Tropical Livestock Genetics and Health (CTLGH), it is timely to assess the impact and legacy of this ambitious (in name as well!) IDEAL project. Therefore, the form of this paper is to collect together the findings and impacts of the IDEAL project, from the authors perspective, and propose how these may have impacted on the stakeholders.

## 2 Materials and methods

The IDEAL project was a longitudinal field study of new born calves carried out between October 2007 and October 2010 in Western Kenya (Bronsvoort et al., 2013). It was funded by the Wellcome Trust and conducted collaboratively by the University of Edinburgh, the University of Pretoria, the University of Nottingham, the International Livestock Research Institute (ILRI), and the Kenyan Department of Veterinary Services. The design of the project is described fully in Bronsvoort et al. (2013) but the key points are described for the reader here.

**Fig. 1:**
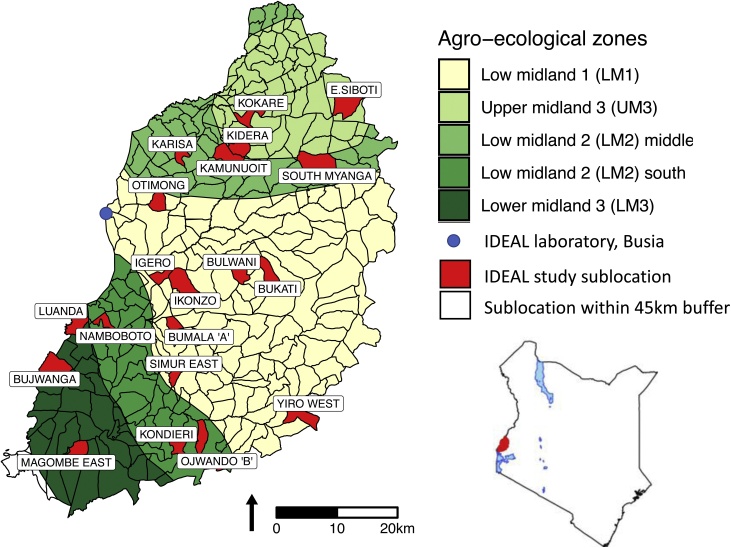
Map of Kenya showing the study area, the 4 agro-ecological zones and sublocations. The sampled sublocations are highlighted. In the LM zones, the annual mean temperature is 21-24 °C, minimum of 14 °C whilst the annual mean temperature in the UM zones is slightly cooler at 18-21 °C (minimum temperature 11-14 °C). Humidity is highest in zones labelled one, and decreases to semi humid in zones labelled three. Cattle can be found in all zones and the main crops grown in each region is as follows: LM1: sugarcane zone; LM2: marginal sugarcane zone; LM3: cotton zone; UM3: marginal coffee zone (FAO, 1996). In the study area, LM2 is split into two by LM1.

During the study, 5337 visits were made to 548 East African shorthorn zebu calves. These were recruited as newborns from 20 sublocations (the smallest administrative unit in Kenya). Daily visits were divided between two field teams comprising either a veterinarian or a senior animal health assistant and at least one other animal health assistant. These sublocations were all within a 45 km radius of Busia town (Fig. 1). This represented the area that could realistically be sampled during daily visits with a return to the laboratory each evening. The study site covered an area extending from Lake Victoria to the foothills of Mount Elgon, thus representing four agroecological zones. Recruitment was achieved through farmers reporting every calving event to their village elder, who informed the assistant chief of their sublocation who in turn contacted the IDEAL project staff. After receiving all the calving reports, between one and three cow-calf pairs were randomly recruited into the study each week. Recruitment was carried out in a five-week cycle with four of the 20 sublocations being visited each week to ensure an even distribution of all sublocations through time, and therefore season, and to enable a logical and efficient work pattern. There were no visits on Fridays allowing the lab team to have the weekend off.

The sample size chosen for this study (a minimum of 500 calves) was based on logistical constraints and the ability to detect a minimum relative risk of 3 with 80 % power whilst allowing for some losses (Bronsvoort et al., 2013). The relative risk was based on one risk factor. In order to be eligible the calf had to meet a set of specific selection criteria which were (1) the calf had to be between 3 and 7 days old at recruitment; (2) the calf was not born as a result of artificial insemination; (3) the dam was not managed under zero-grazing conditions (as this is likely to reflect cattle associated with dairy production and potential exotic genetics and thus not be representative of the traditional small holder farming system); and (4) it did not have any congenital deformities (as we were only interested in infectious causes of disease and the association with host genetics rather than direct genetic diseases) (Bronsvoort et al., 2013).

Farmers were compensated at a rate agreed with the staff of the District Veterinary Office; this comprised the estimated cost of raising the calf for one year as calves were nominally owned by the project for that year. Calves were raised according to the farmer’s practices. However, if the calves became ill then they were treated by and at the discretion of the project vet. Veterinary products were administered to project calves to ensure welfare, but after such treatment calves were dropped from the study and their data censored.

Data were collected about the farm, the farmer, the dam and other animals on the homestead. Each calf was visited every five weeks from birth until 51 weeks of ages, and a clinical examination was conducted at each of these visits. Interim visits also occurred if clinical episodes were reported by the farmer, and a post-mortem was carried out if the calf died. Blood and tissue samples were collected in association with all visits and screened using a range of diagnostic tools. Growth data, clinical data, haematological values, and post-mortem examination of animals that died while participating in the study formed the outcomes of interest.

During the period of fieldwork, prior to being placed into the biobank, collected samples were tested for a limited number of parasites and other infectious organisms in a field laboratory. Pathogen identification during the field collection phase concentrated on what was possible, what was probable, but also on what was affordable, and consequently represented mainly those pathogens that could be observed through light microscopy or using commercial ‘penside’ kits. Faecal samples were unable to be stored, but all tissue and blood samples were recorded into the biobank.

The data gathered during the field phase of the study were initially stored in a Microsoft Access database which was designed for easy and secure collection of information. It was subsequently transferred to a relational MariaDB^2^ database that could be accessed and updated remotely giving all staff access to the data for analysis. The data were held in 217 tables in a relational database format.

In 2019, the data in the MariaDB database were aggregated into seven tables and a Python web application was developed (Callaby et al., 2020). This application includes a custom search form which enables users to query the data in a user-friendly way without writing code. Thus, the IDEAL data are now accessible through an open access web-hosted database: http://data.ctlgh.org/ideal/. This database links to the biobank of samples, providing a resource for researchers who had no original involvement with the project. The website has request options, which provides a method for interested researchers to request access to the biobanked samples.

## 3 Results and discussion

The IDEAL project has allowed us to generate a dataset that is unusually comprehensive, not just for cattle but for any host in any setting. Information gathered during the project was used to provide baseline information on prevalence of pathogen exposure in calves in states of health and disease. Some of the identification of infection represented clinically silent events, such as seroconversion or evidence of parasitaemia. Others were clinically observable such as mortality, clinical syndromes and poor growth. Below we discuss the results of the IDEAL project in terms of the possible impact it had on each of the stakeholders (the animal, the farmer, the consumer, the policy maker, the funding body, and the researcher and their institution); the epidemiological challenges and the logistical lessons we learnt as a result of running such a study are also discussed. It should be noted that this project was designed as a research project and that farmer education and community outreach were not the primary objectives of the project.

### 3.1 The impact on knowledge and the research community

Over 50 different pathogens (or evidence of exposure to pathogens) were initially identified during the project (Bronsvoort et al., 2013). Haemoparasites and gastrointestinal parasites were the predominant finding, with nearly all the cattle infected with these agents in their first year of life. The longitudinal data allowed us to more definitely associate these identified pathogens with incidences of disease and production loss, and to demonstrate where infection / exposure had occurred without such impacts. Certain pathogens showed significant impact at the population level, notably *Theileria parva* (East Coast fever, ECF) and *Haemonchus placei* (haemonchosis), these being the main causes of death for 33 (6%) and 10 (1%) calves respectively (Thumbi et al., 2014). However, the data demonstrate that 382 calves did not succumb to *T. parva* infections although infection was detected during their first year.

This variability in calf performance was investigated through time, and again attributed to different infections because of the regularity of weight measurement in the calves. The study design enabled estimates to be made on the impact of co-infections on survival, health, productivity in the form of growth, and on haematological variables. The two ‘common killers’ were shown to be more likely to lead to mortality if accompanied by other factors; the presence of other infections, the genetic composition of the individual, and life history traits of the calf such as certain husbandry practices (Bronsvoort et al., 2013; Murray et al., 2013; Thumbi et al., 2013b, 2014).

As *T. parva* was the most significant cause of mortality, it was of interest to identify differences between those calves that did and did not die from the infection, not least because, as mentioned above, almost every individual in the cohort was exposed. This became the focus of the co-infection and mortality work. It was identified that the presence of *Trypanosoma* spp. in the life of the calf was associated with a 6 times (95 % CI = 1.4–25.8) increase in the hazard of death (Thumbi et al., 2013b, 2014). In addition, those calves that had higher faecal worm egg outputs at the visit before death were also more likely to die when infected with *T. parva* (Thumbi et al., 2013b, 2014). An increase in strongyle eggs of 1000 eggs per gram of faeces was associated with a 1.5 times (95 CI = 1.4–1.6) increase in the hazard for ECF mortality (Thumbi et al., 2014). The mechanism by which this happens is unclear, but a similar co-infection profile has been identified in *Plasmodium* spp., also a protozoan parasite, and for hookworm helminth infections in humans (Brooker et al., 2007).

A significant and possibly clinically relevant interaction was identified between prior seroconversion to *T. mutans* and a reduced risk of death following a subsequent infection with *T. parva* (Woolhouse et al., 2015). Calves with previous exposure to the less pathogenic species of *Theileria* (*T. mutans*) experienced an 89 % (95 % CI = 47–99 %) reduction in mortality associated with a subsequent *T. parva* infection (Woolhouse et al., 2015). This effect persisted when controlling for age at infection. This finding triggered a number of further investigations following the conclusion of the IDEAL project (more below).

In addition to mortality, *T. parva* also impacted on production through a decreased growth rate. However, when cattle were co-infected with *T. parva* and *T. mutans* they experienced a relatively higher growth rate compared to when they were only infected with *T. parva* as a single infection (Thumbi et al., 2013a). In contrast, when cattle were infected with *T. parva* and *Anaplasma marginale* they experienced relatively lower growth rated compared to either infection on its own (Thumbi et al., 2013a).

Infection with gastrointestinal worms, especially increased burden (assumed from increased faecal worm egg count (FWEC)), was unsurprisingly found to be associated with decreased productivity. Losses were estimated to result in a 3.3 % decrease in growth rate (Kg/day) for every 1000 increase in Strongyle eggs per gram of faeces (Thumbi et al., 2013a). *H. placei* is believed to be the dominant species in this context (partially confirmed by larvation carried out by the study) and therefore it was not surprising to find that worm burden was correlated with changes in blood parameters (decreased packed cell volume, white blood cell counts, and total serum protein (van Wyk et al., 2014; Callaby et al., 2015)).

Despite the intense infectious disease pressures, the majority of calves passed through their first year of life without clinical disease being observed (n = 295, 54 %), and a minority of calves experienced the majority of clinical episodes, with 24 calves having 3 or more clinical episodes (Jennings, 2013). Multiple clinical episodes were apparently related in time, suggesting that they were due either to the same or connected pathogenic processes (Jennings, 2013). A low birth weight, larger herds, and older farmers were all risk factors for being a sick calf. Both high helminth burden and *T. parva* were found to be significantly associated with clinical disease at a population level (Jennings, 2013).

Furthermore, a lot of variation was seen in the clinical presentation of disease (Jennings, 2013). The clinical signs associated with ECF, were found to be very variable. Although this may have been partly due to the varying times in the disease process that calves were observed prior to death, the complication of the clinical picture was also suggested to be due to co-infections.

In addition to examination of the impact of infectious diseases, IDEAL also allowed the Kenyan Veterinary Department to survey the husbandry practices common in the region. The random stratified sample selection provided a non-biased representation of small-holder farms keeping cattle in the region, where this would be the predominate farming system. The information collected from the cattle owners demonstrated there was a lack of provision of housing for cattle, minimal use of veterinary services, and lack of planned breeding (Bronsvoort et al., 2013). These factors in addition to infectious disease pressures were all limiting the potential of livestock production in this region (Bronsvoort et al., 2013; Thumbi et al., 2013a, b; Toye et al., 2013; Thumbi et al., 2014). For example, the use of supplementary feeding, whereby crop residues were offered to the calves left at the homestead when adult cattle went to graze on the fields, reduced the hazard of haemonchosis deaths by 90 % (95 % CI = 48–98 %) compared to those calves which did not receive supplementary feeding (Thumbi et al., 2014). It is likely that supplementary feeding reduces exposure to helminths as well as improving the calf’s nutrition so the effects of helminthosis are reduced (Thumbi et al., 2014).

IDEAL also investigated the association between calf genotype and phenotype, mostly focused on the expression of clinical disease associated with infection, but also to describe the population, and the breeds that had contributed it. Initially, all the calves were genotyped using a 50K SNP-chip. The IDEAL calves were purposively recruited to be the indigenous East African shorthorn zebu breed and the genetic analysis showed the majority of these animals were genetically homogenous with an admixed genetic constitution of 84 % Asian zebu and 16 % African taurine ancestries (Mbole-Kariuki et al., 2014). However, 123 individuals revealed evidence of recent introgression with European taurine breeds, which is thought to be the legacy of abandoned breeding programmes which happened in the area (Mbole-Kariuki et al., 2014). Significantly, these European taurine introgressed animals were more likely to experience clinical illness (OR = 2.4, 95 % CI = 1.5–3.8, Murray et al. (2013)). In addition, 37 animals were found to have low levels of heterozygosity (i.e. inbred animals), and these were more likely to die or experience clinical illness (OR = 3.5, 95 % CI = 1.05–15.1 Murray et al. (2013)).

The IDEAL biobank (currently held at ILRI in Nairobi) has allowed on-going analysis of samples. A subset of IDEAL cattle was genotyped with the Illumina BovineHD Genotyping BeadChip. These genomes were integrated with other bovine genomes collected from across East Africa to allow the identification of candidate regions of positive selection. This selection analysis identified regions that were postulated to have been selected to increase fitness with respect to reproduction and survival. QTLs associated with these traits (e.g. reproduction, immunity, and heat tolerance) have been identified as targets for future breeding programmes (Bahbahani et al., 2015, 2017).

The samples have also been used in projects which were not envisaged when the project was conceived. For example, genotyping of the DNA from a cohort of the animals, together with the clinical records, has been used to extend the findings from a separate study aimed at elucidating the genomic basis of tolerance to ECF (Toye et al., pers. comm.). The samples will also be key to validating a deep sequencing approach to determining the haemoparasite burden within the host, which will prove invaluable in future epidemiological studies.

In addition to the research outputs from the project, the training legacy of the project is substantial and significant. IDEAL has produced six PhD theses, four MScs by research and two members of support staff were supported to complete a taught master’s programme. All students have remained in science. We regard this as a significant contribution to training the next generation of tropical animal health researchers.

Lastly, the collaborations developed have been invaluable, and they continue in new projects. For example, two of the IDEAL PIs are the co-leads of the ‘Animal Health and Genetics Program’ of the CTLGH. The data and knowledge generated by the IDEAL project have helped a number of other studies to start, based on the information and methods gained from the study. As mentioned above, the finding of heterologous protection to *T. parva* induced by co-infection with *T. mutans* is the subject of a Bill & Melinda Gates Foundation-funded study to assess the feasibility of using *T. mutans* as a commercial alternative control method for ECF. Furthermore, two major projects, led by Professor Eric Fèvre of the University of Liverpool and ILRI, have also been undertaken in Busia which build on the results and infrastructure from IDEAL. The PAZ project (People, Animals and their Zoonoses, www.zoonotic-diseases.org/project/paz-project/) was targeted at endemic, neglected zoonoses in livestock and humans, and the impact of co-factors on the epidemiology of, and burden imposed by, these diseases. Whilst, the ZooLinK project (Zoonoses in Livestock in Kenya, www.zoonotic-diseases.org/project/zoolink-project/) seeks to enable Kenya to develop an effective surveillance programme for zoonotic diseases, integrated across both human and animal health sectors. The field activities of both projects were centred around the Busia laboratory, originally established by the IDEAL project.

Since the conclusion of the IDEAL project, Kenya has moved to a decentralised system of Government, with counties being the governing unit. The County Government of Busia, together with ILRI, is working towards commissioning Busia as a Government surveillance site, which would extend the laboratory’s impact into new areas.

### 3.2 The impact for policy makers

In terms of impact for policy makers, IDEAL showed that it was possible for a project to set up and maintain a functional laboratory and keep it running in rural Kenya. As previously described, this laboratory is still being used by other projects, thus it has become an important legacy and has also enhanced the diagnostic capacity within the region.

Yet, the funding of the laboratory raises issues in the scope of ‘research for development’. Since about 2007, the laboratory has been financed almost entirely by funds from various research projects. The projects have demonstrated the feasibility of having such a laboratory in a relatively remote area and the benefits to be derived from it. All of the equipment in the laboratory was left to the government through the DVS after the conclusion of the project. It does however raise the issue of sustainability. While researchers should seek, and are seeking, funding to make further use of the laboratory to address critical research issues, a more sustainable outcome is clearly needed for a research funder to maintain the basic running of the laboratory and encourage researchers to make use of it both during the project operation but also after the close of initial activities. At the same time, it is ultimately the decision of the local authorities and policy makers whether to maintain the laboratory for national research activities or for more routine diagnostic and epidemiological activities. Although not a development project, in retrospect, more could have been made of the laboratory as an outreach centre during the operation of the project. This region is dependent on livestock. Having the access to diagnostic facilities would be of value. The project legacy developed the capacity of animal health technicians in this region to provide such a service and this had the potential to be capitalised on after the project was finished.

Lastly, in terms of impact on policy makers, IDEAL was funded as a research project not a development project and so training of the farmers and extension was not part of the funded grant. Nevertheless, an end of project meeting was held with the Kenyan agricultural department and the veterinary office. This provided the initial findings to policy makers, potentially offering evidence or context to their work. The results from the study have been published, in the main, in open access formats, and this provides those without institutional access to journals the opportunity to use the knowledge for policy or project development.

### 3.3 The impact on calves and their owners

Findings such as the effect of breed improvement programs on the genetics of livestock within the region have a direct impact upon calves and their farmers, since IDEAL showed little evidence of success at the attempts to upgrade local cattle (for improved milk production) with exotic European breeds. Local breeds are adapted to the infectious diseases found in this environment. In contrast, evidence from this study as well as others, suggests that, without significant investment in housing and parasite management, exotic breeds do not thrive and therefore become unattractive in a low-input environment. Furthermore, exotic breeds been shown to be more susceptible than local breeds to some infectious diseases that are prevalent in Africa, such as ECF, bovine tuberculosis, and trypanosomiasis (Roberts and Gray, 1973; Coetzer and Tustin, 2004; Glass et al., 2005; Ameni et al., 2007), as well as requiring high inputs to support their production potential. Although many call for introduction of exotic breeds to the East African context, evidence suggests that such investment is often rapidly lost from the population. In addition, there is a risk that if exotic breeds are introduced without care it will result in the loss of the unique adaptive traits present in the local breeds. The value of this genetic resource must be protected (Mwai et al., 2015).

However, there seems to be potential for improvements through selection from within the local breeds. For instance, there was huge variation (10-fold) in growth rates of the calves and so it is likely other productivity parameters such as milk production are equally variable (Bronsvoort et al., 2013; Lesosky et al., 2013; Thumbi et al., 2013a). This variation is likely to be due to inputs, but may also represent opportunities for genetic selection. However, data from this project, concluded in 2010, suggests that farmers in this area were not at that time engaging even in planned bull selection. The possible reasons for this are various and could be logistical, cultural, or driven by market. Therefore, before efforts and investment are made in the development of, for example, breeding of bulls for artificial insemination, there will need to be better understanding of the barriers to planned breeding, and this can only be done through embracing the methods and expertise of social science. The IDEAL project provided the prevalence of practices, and this data should be utilised now to further investigate practice and possibilities for sustainable change towards positive management techniques, that may include genetic improvement.

Analysis showed that strongyle egg count was heritable (h^2^ = 23.9 %, SE = 11.8 %, Callaby et al., 2015). In addition to this, calves that were born small were also found to have higher burdens (if FWEC can be assumed to represent burden) of strongyles. It has been demonstrated in several other contexts that worm burden is partially determined by genetics, and that it is possible to select animals that are resistant to gastrointestinal nemotatoes. Selection for this trait, will decrease the FWEC, and thus also result in a decrease in pastural contamination, leading to additional benefits for all the animals grazing the same pasture (Bishop, 2012). However, the same barriers will confront programmes that aim to utilise genetics as a means of disease impact reduction. Genetic improvement relies on both systems for dissemination of the genetic material and an ability to measure the phenotype of the female breeding stock on farm.

East Coast fever was demonstrated to be having an impact on production in this region, and consequently the finding of a possible protective effect of a co-infecting parasite generated a lot of interest. and has generated further, on-going research. In addition to ‘protective’ effects, synergistic effects i.e. infection with one pathogen making the effect of another more serious, were also identified. Exploitation of this knowledge has the potential to produce relatively easy and sustainable interventions. For instance, the mortality rate from ECF was higher in animals which had high strongyle worm burdens (Thumbi et al., 2014). In other words, de-worming could have the added benefit of reducing mortality due to ECF. The communication of this message may encourage farmers to engage in the worming of younger calves. However, this must be approached in a sustainable way, and will currently be limited by the limited access to expert animal health advice and diagnostics.

In terms of disease awareness, IDEAL found that there was a mismatch between farmers’ perception of the risk and reality of disease threats (Bronsvoort et al., 2013). In response to IDEALs questions about the risk of disease to their cattle, the farmers tended to focus on high impact transboundary diseases like Foot and Mouth Disease. Yet, IDEAL showed that it was the chronic endemic illnesses such as high worm burdens which were having a bigger impact on the cattle in the long term. Perry and Grace (2009) state that control of a high impact disease does not guarantee increases in productivity or profitability. Findings from the IDEAL project suggest that treating cattle for the chronic conditions, especially in a system where international export is not a significant part of the market, could lead to greater economic rewards for farmers and likely welfare improvements for livestock. It is important to note, that the project concentrated on those diseases affecting young stock but farmers were asked to report their perceptions of disease risk for their herd, which may lead to this mismatch. Farmers in the region would benefit from training and education in the disease threats facing their herds and how to recognize them, the losses that these diseases may cause, and the treatments and preventative measures available to reduce those losses. IDEAL trained local animal health assistants and employed technicians from local institutions, thus building veterinary capacity in the region, and the hope is that this contributed in a small way to animal health and the local rural economy, and this expertise could be utilised by the government to support rural development.

As a small side project, and one that offers solutions in resource poor settings, IDEAL developed an algorithm that allows producers to accurately estimate the weight of their animals using a heart-girth measure (Lesosky et al., 2013). This tool is commonly used by farmers in other settings, but the tapes are designed for use in Holstein-Friesian breeds and are not appropriate for use in the smaller shorthorn zebu breed. This tool developed as part of the IDEAL project, allows for accurate measurement of production, for accurate dosing of pharmaceutical products to stock, and for estimation of value when marketing (Lesosky et al., 2013).

Finally, it must be noted that all the knowledge gained through the IDEAL project remains useless if it remains in research silos. It only becomes useful when accessed by policy makers and NGOs working in this area, and by interdisciplinary teams of researchers. Local meetings in Busia were arranged in the aftermath of the project and major findings were presented to animal health assistants. As calves left the study farmers were provided with a report of the pathogens that had already been identified to have infected their calves. This mainly comprised the gastrointestinal and haemoparasites. It is timely to consider how the new and more integrated knowledge can be presented and used by stakeholders.

### 3.4 Overall impact for calf production

**Fig. 2:**
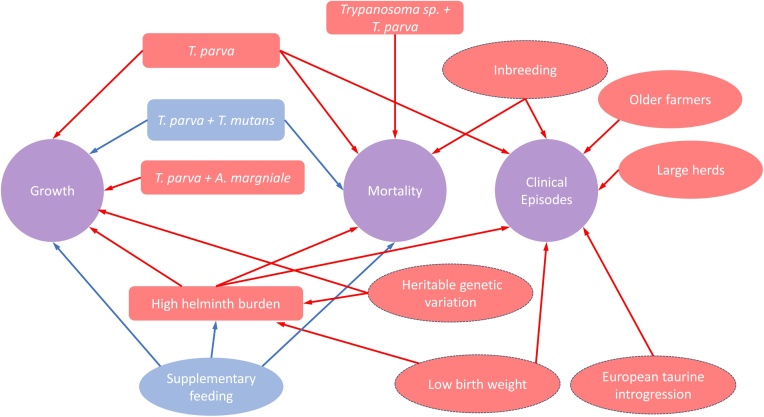
Diagram of all the infectious (rectangular boxes), host (oval outlined boxes) and husbandry (oval no outline boxes) factors which affect calf growth mortality and clinical episodes described in this review. The arrows show the variable which the factors impact upon. Factors which increase the risk or have adverse effects are shown in red and those factors which decrease the risk or have positive effects are shown in blue.

The above review shows how the presence of infections, the genetic composition of the individual, and life history traits of the calf all influence the host outcome in terms of growth, mortality, and clinical disease. This review is represented in the schema shown in Fig. 2 which attempts to draw all this information together and symbolises the positive and negative effects of these factors on the calf in terms of growth, mortality and clinical illness. Fig. 2 consists of a multitude of factors, which all interact and form a complex web. For simplicity, this figure includes the headline interactions that involve few actors. There are many more associations have been accounted for in the papers produced by the IDEAL project and used to inform the relationships observed in this schema which have not been illustrated in the figure due to their complexity. This schema provides visualisation of how comprehensive datasets like that generated by the IDEAL project can begin to describe and untangle these complex relationships.

### 3.5 Logistical lessons

The IDEAL project proved to be a tremendously challenging project. Below we highlight the five main issues we faced in running the project and solutions we took to overcome them.

#### 3.5.1 Setting up and running the field study

Firstly, there were logistical challenges in setting up a field laboratory, and running sophisticated laboratory equipment despite poor electricity and water provision, lack of internet and a long supply chain. In order to achieve this, the project refurbished and equipped a Kenyan Directorate of Veterinary Services (KDVS)-laboratory in Busia. All the microscopy and faecal culture was carried out here, and for more complex diagnostic tests, especially those that required DNA clean rooms, samples were transported to ILRI and the University of Pretoria. These issues were anticipated and planned for. For example, to mitigate losses of samples whilst they were being transported to Nairobi, samples were taken in duplicate and only one of the duplicates was transported at any one time. Furthermore, biobanking of the material has allowed more diagnostic tests to be carried out as need has arisen or as new tests have become available.

#### 3.5.2 Recruitment and monitoring of cattle

There were similar challenges in setting up the recruitment and monitoring of calves from such a young age (time critical recruitment) over a wide area with poor accessibility (bad roads, floods, etc.). Data collection commenced at a time when mobile phone ownership was becoming common in the study region. Hand held data recording devices were available and affordable, and towards the end of the data collection period, broadband internet arrived in Kenya. In current times, the use of apps such as Open Data Kit (ODK, Hartung et al., 2010) have helped to ease and simplify these challenges, making data collection easier.

Even with the frequent visits and extensive visit protocol, it quickly became apparent that the 5 weekly sampling strategy was too long to pick up the fine scale dynamics of infection. In the initial stages of the project a group of calves was visited weekly, but this was abandoned early on as it was realised that such an approach would be unsustainable without substantial and impossible levels of investment in more field teams (people and vehicles). This has meant that much of the clinical change (other than long term impacts such as growth or permanent outcomes such as death) went largely unobserved. In contrast to the host outcome data, defining the phenotype from an infection status point of view has become both easier and cheaper in recent years and, because of the biobank, will allow further enrichment of the data. For example, the development of new diagnostic tools, such as the ‘haemobiome’ offers quantitative identification of many haemoparasites from a single ‘test’. Such high-resolution phenotypes offer the potential to understand the dynamics between coinfecting pathogens (Hemmink et al., pers. comm.). This can be linked with the phenotype and genetic data already gathered, further enhancing the questions that can be asked.

Looking back at the local legacy, IDEAL used a structured reporting system for calf births where the farmers reported every calving event to the village elder, who informed the assistant chief of the sublocation who in turn contacted the IDEAL project staff. By involving the whole community in the project in this way IDEAL could successfully engage local stakeholders and recruit individuals into the study, and encourage community buy-in.

On reflection, one of the investments made by IDEAL that had most value for successful field data collected was a several months long dry run of the field study. This added to the duration and expense of the project, but it paid off many-fold by identifying issues and, at the same time, giving the project team the most effective possible training. It also provided an opportunity to pilot and validate questionnaires, protocols, and calf identification processes. We would recommend repeating this exercise for similar future projects. Furthermore, a laboratory management workflow list was generated from the visit automatically and this ensured that laboratory staff were able to efficiently manage their work load and avoid mistakes. Investment was made at the start of the project to ensure that the systems were robust, and once the project was in the main recruitment phase this system was reliable.

#### 3.5.3 Unanticipated and unplanned for events

The project period encompassed a period of unexpected turmoil in Kenya when post-election violence erupted across the country in 2008. IDEAL also had to contend with these unanticipated events. This is one of the challenges of such study designs; the power of the longitudinal study can be affected by external events, with the gaps caused by the unrest evident in the dataset. Not only were a number of routine visits missed, but clinical events passed unnoticed leaving the phenotypes of at least six calves incompletely described as they died unobserved by the project.

Unplanned for logistical failures were also burdensome for the project, both because of the cost of repair, but also because failure of infrastructure had the potential to lead to loss of samples. This was costly in terms of both money and time. Weekly teleconferences are therefore an essential part of project management.

#### 3.5.4 Diagnosing the cause of death

Post-mortems were carried out under difficult conditions, and around half way through the project a post-mortem facility had to be built to avoid the risk of contaminating the areas around the government building with potentially infected material. Gross post-mortems were recorded in a standardised way and analysed locally. However, final diagnosis was delayed until completion of the study to allow sensible work flows. Despite substantial investment in testing and man-hours spent pulling together diagnostic information, in 29 % of cases it was not possible to assign a cause of death. This is not unusual. For example, for those cases presented for abortion to the Animal and Plant Health Agency (APHA) and Scotland's Rural College (SRUC) veterinary surveillance centres in 2017, 13 % remained undiagnosed (Animal and Plant Health Agency, 2018).

#### 3.5.5 Database management

Finally, database management on this scale is a significant challenge and preparing such a database to even minimal open access standards needs its own budget. It is only due to the significant amount of time and funding that, 10 years later, this database is ready to be opened to the public (http://data.ctlgh.org/ideal/). Converting the data from a form that allowed it to be efficiently collected (resulting in over 217 interlinking tables) to a form that allows it be efficiently queried has been a major and specialist undertaking (Callaby et al., 2020). One of the lessons learnt from this project has been the need to plan for that and invest in expert advice in the earliest stages of the process.

### 3.6 Epidemiological challenges

The main advantage of the IDEAL project was its ability to understand complex relationships which would not have been possible to do with a smaller more defined study which is focused on one pathogen. However, from the epidemiological perspective there are also limitations with the IDEAL project, principally around the trade-off made between the depth and the breadth of the study. IDEAL was designed to addressed broad questions, focusing on the estimation of the incidence of multiple diseases. To achieve this the IDEAL project recruited 548 calves as this was the maximum number of cattle the field and laboratory teams could manage to track and test for multiple diseases every 5 weeks, within the given budget. However, this resulted in the study not having enough calves needed for population level genetic association studies to look at genetic effects in detail. Therefore, a key lesson learned from the project is the need to achieve a balance between a large enough population to provide statistical power for genetic analyses, with one that is small enough to obtain detailed clinical information and sampling at a suitable frequency.

Since the end of IDEAL, the costs of sequencing have fallen dramatically and new diagnostic techniques have been developed which offer future projects that follow IDEAL the opportunity to carry out more detailed testing and investigation in real time, using one test. Decreasing the costs may enable deeper questions to be asked about the population and reduce this trade-off. In addition, the decreasing cost of testing will allow for similar projects to focus on multiple geographic locations or farming systems and thereby increase the impact of any findings.

### 3.7 The future of the IDEAL project

Data generation from samples obtained during this ambitious longitudinal study still continues and is driving further research, enabled by the launch of its extensive open-access database and biobank (http://data.ctlgh.org/ideal/). As shown above, the project has also led to the development of numerous other studies, particularly focusing on the control of ECF. In addition, a new haemoparasite typing tool developed from the IDEAL samples, the ‘haemobiome’, now offers the research community a method to quantitatively identify many haemoparasites from a single procedure. The findings from these higher resolution phenotypes for each calf will be published soon (Hemmink et al., pers. comm.). Used in combination with new high-density SNP genotyping of the IDEAL cattle, this information will drive deeper understanding of the coinfections occurring within the region and allow us to identify novel genomic markers for growth, disease resistance and survival.

## 4 Conclusion

In summary, IDEAL showed that the cattle existed in conditions of heavy infectious disease pressure and minimal investment in prevention or treatment. Although most calves experienced multiple infections by potentially fatal pathogens, more than 80 % survived until 51 weeks of age, and approximately 50 % of animals were not observed to suffer clinical illness. Coinfections with other pathogens partly determined whether or not calves experienced clinical disease. Moreover, a significant fraction of mortality and ill health was attributable to adverse effects of inbreeding and European taurine introgression in affected calves. There was variation in growth rates but, nonetheless, some calves managed impressive weight gains despite multiple infections and living in a harsh environment.

In terms of study design, the real value of IDEAL has come in the effort that was taken at the outset to ensure the long view. Availability of mobile connections, the careful use of sample workflow models, and fastidious attention to detail led to early systems designed to build resilience and longevity. The database and bio-bank has allowed the legacy to grow and it has proven to be a worthwhile investment, forming one of the major assets of the CTLGH. It has also allowed us to begin to better describe the contributors to the difference in calf outcome, and is leading to the development of interventions that have the potential to improve animal health and productivity, such as the work investigating ECF and that investigating the genetic drivers of success in cattle in this context.

## Authors’ contributions

RC and AJ wrote the manuscript; MW, BB, OH, JW, and PT. designed and co-led the IDEAL project; STM, AJ, MN, IC, conducted the field work, participated in the development of the diagnostic tools and interpretation of the results; HK and PT were responsible for diagnostic testing; RC was responsible for the transformation of the open access database. All authors read and approved the final manuscript.

## Funding

This research was funded in part by the Bill & Melinda Gates Foundation and with UK aid from the UK Government’s Department for International Development (Grant Agreement OPP1127286) under the auspices of the Centre for Tropical Livestock Genetics and Health (CTLGH), established jointly by the University of Edinburgh, SRUC (Scotland’s Rural College), and the International Livestock Research Institute. The findings and conclusions contained within are those of the authors and do not necessarily reflect positions or policies of the Bill & Melinda Gates Foundation nor the UK Government.

## Ethics

The IDEAL project was reviewed and approved by the University of Edinburgh Ethics Committee (reference number OS 03−06) and also by the Institute Animal Care and Use Committee of the International Livestock Research Institute, Nairobi. Standard techniques were used to collect blood and faecal samples for diagnosis and identification of disease and infecting pathogens. The calves were restrained by professional animal health assistants or by qualified veterinary surgeons. A veterinary surgeon was available to examine any calf falling sick during the course of the study. Any calf that was in severe distress due to trauma or disease was humanely euthanized by intravenous injection of sodium pentobarbital by a veterinary surgeon. All participating farmers gave informed consent in their native language before recruiting their animals into the study.

## Footnotes

2 free and open source fork of MySQL.
